# Concomitant Laparoscopic Burch Urethropexy and Combined Vaginal-Laparoscopic Mesh Sling Removal (x2) for Pain and Persistent Stress Urinary Incontinence

**DOI:** 10.1155/2016/6180756

**Published:** 2016-10-31

**Authors:** Sarah A. Huber, LaChanda Dunlap-Wright, John R. Miklos, Robert D. Moore

**Affiliations:** ^1^International Urogynecology Associates of Atlanta and Beverly Hills, Alpharetta, GA, USA; ^2^Philadelphia College of Osteopathic Medicine, Georgia Campus, Suwanee, GA, USA

## Abstract

Although midurethral mesh tape slings are considered the standard of care in the treatment of female stress urinary incontinence (SUI), complications such as pain, dyspareunia, or erosion are known to occur in addition to persistent incontinence. The management of these types of mesh sling complications can be very complex, especially when the pain is not just isolated to the vagina but extends into other areas, such as the abdomen which requires a much more extensive dissection. Additionally, if a mesh sling needs to be removed, the patient will most likely have a return of her SUI that often necessitates subsequent treatment. Vaginal and/or laparoscopic removal or revision of mesh tape slings should be considered in patients presenting with complications such as vaginal pain, abdominal pain, dyspareunia, or urinary obstructive symptoms. In those patients who demonstrate persistent SUI, concomitant laparoscopic Burch urethropexy can be considered and can safely be performed at the time mesh removal. In this case report we present a patient who required a dual-approach removal of two painful midurethral slings in addition to concomitant treatment of persistent SUI with a laparoscopic Burch urethropexy procedure.

## 1. Introduction

Over the past 20 years, the midurethral sling (MUS), which includes the single-incision minisling, tension-free vaginal tape, and transobturator tape, has become a popular and evidence-proven option for management of bothersome stress urinary incontinence (SUI) in women who desire surgical management. This is due in large part to their ease of application, minimally invasive approach, and comparable outcomes to other more involved procedures such as Burch urethropexy [1, 2]. However, no surgery is without the potential for short or long term complications to varying degrees. Unfortunately, for the mesh sling procedure, its complications have acquired widespread media and public attention. In 2008 and 2011, the Food and Drug Administration (FDA) issued warnings regarding the safety of vaginal mesh, indicated for prolapse or to a lesser extent SUI based on identifiable risks for mesh erosion, pain, infection, and failure [3, 4]. Based on their independent literature review and investigation of reported incidents, they identified the main adverse outcomes with SUI slings to be pain followed by erosion through the vaginal mucosa at around 2% prevalence [5]. Other complications included dyspareunia, bladder injury, nerve injury, urethral or bladder erosions,* de novo* urgency, urethral obstruction with voiding dysfunction, and unresolved SUI [6, 7]. Additionally, the failure rate of MUS, specifically recurrence or worsening of SUI, is approximately 10%, comparable to other anti-incontinence procedures [1]. Often women who present with a chief complaint of pain or erosion also express frustration due to persistent SUI.

Approximately 50% of women who present with symptomatic sling complaints require surgical management after failure of less invasive methods [8–10]. When conservative measures have failed to adequately treat a complication, surgical removal or revision of the MUS has been proven to provide relief of symptoms associated with some of the aforementioned mesh-related complications. However, often this leaves the patient with recurrent or worsening SUI. Typically, treatment for SUI is not addressed during the initial surgery for sling removal and is delayed until after completely healing from the revision due to concern for inflammation, blood loss, or an anticipated delay in anatomical restitution following sling removal. However, a delayed secondary surgery for anti-incontinence is not without its own risks, particularly complications surrounding adhesions and scarring that can obscure the retropubic space after previous dissection for traditional bladder suspension or for retropubic mesh sling removal [11].

In the present case we describe the successful treatment of recurrent SUI in a patient requiring vaginal/laparoscopic removal of two midurethral slings with concomitant laparoscopic Burch urethropexy.

## 2. Case Report

A 50-year-old postmenopausal para 3 woman with a prior single-incision minisling (AMS, Minnetonka, MN) and subsequent retropubic (TVT, Gynecare, Somerville, NJ) sling presented with complaints of severe dyspareunia and chronic pain. On initial interview, she also reported persistent refractory stress urinary incontinence (SUI). Her past medical history was significant for depression, anxiety, and hypertension for which she was taking a low dose of hydrochlorothiazide. Her other prior surgeries included a cholecystectomy and tonsillectomy. She admitted to a 32-pack-year smoking history and had a BMI of 34. She had never been on hormone-replacement therapy and was not currently sexually active secondary to pain.

Her prior clinical records and operative reports were obtained. Six years before, she had a total abdominal hysterectomy, posterior colporrhaphy, and single-incision minisling for SUI using the standard technique. However, she continued to report persistent SUI and five years after her initial sling placement she received a retropubic (RP) TVT sling placed via the standard technique. Postoperatively, she complained of urinary retention and obstructive voiding, and the RP sling was transected in the midline with relief of some of her obstructive symptoms one month after placement.

Approximately 4 months following her RP sling insertion, she began to experience notable diffuse pelvic/perineal pain localized to the periurethra, groin, right and left lower abdominal quadrants, and suprapubic area. She also experienced dyspareunia as well as persistent mixed urinary complaints. Her specific urinary symptoms included urinary urgency/frequency, nocturia, difficulty emptying her bladder, and stress urinary incontinence. She also reported anal incontinence with need for splinting for defecation. Her preoperative IIQ-7 score was 52.3 and the UDI-6 score was 54.2.

Physical examination revealed contraction and banding of the mesh tape sling(s) bilaterally in the periurethral area. Palpation of the mesh reproduced her pain, with radiation to her groin and up into her lower abdomen, right greater than left. There was no evidence of mesh extrusion. Her exam also diagnosed a stage II anterior and posterior wall prolapse without significant tissue atrophy. She had pronounced urethral hypermobility and a positive standing cough stress test. Urinalysis was negative with prior urodynamic testing showing urodynamic SUI without significant detrusor overactivity or impaired voiding and a normal bladder capacity. Her ICS-IUGA mesh complication score was 1Be/T4/S2, S4 [12].

Based on the patient's history and physical examination it was recommended that she undergo vaginal removal of the single-incision sling and vaginal and laparoscopic removal of the RP mesh tape sling with concomitant laparoscopic paravaginal repair, posterior repair, and Burch urethropexy. All procedures risks, benefits, cure rates, complications, and alternatives to surgery were discussed in detail with the patient. She agreed to the plan of care and informed consent was obtained.

**Figure 1:**
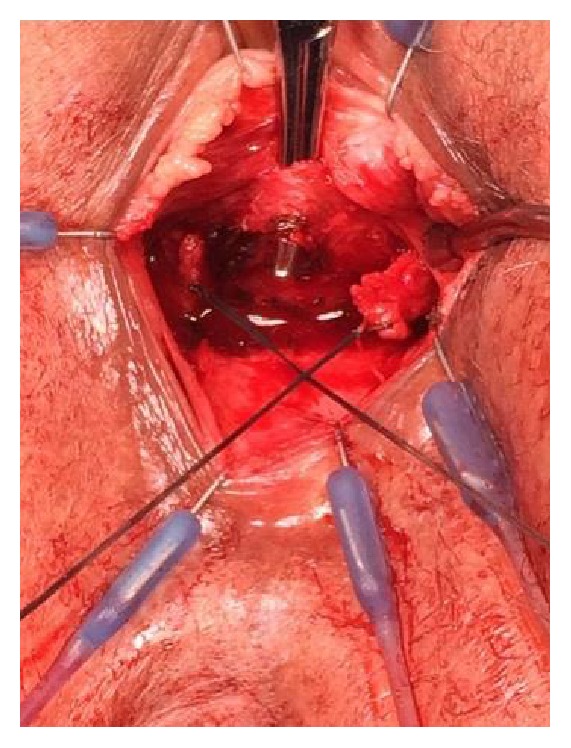
Tagged vaginal arms of transected TVT sling. The minisling has been dissected away from the suburethral tissue and isolated with a right-angle clamp prior to cutting in the midline. Permission to reprint from Miklos and Moore.

Under general anesthesia, a suburethral vertical incision was made and the vaginal epithelium was dissected laterally off the underlying urethra and periurethral tissues. The arms of the RP sling had retracted bilaterally; therefore the SIS was immediately visible. It was carefully dissected away from the urethra, cut in the midline, and then dissected out laterally to its entry into the obturator internus muscle bilaterally. The entire SIS sling was removed intact including its self-fixating tips. The defects in the muscle and fascia were then closed with absorbable polyglycolic acid suture. The edges of both arms of the retropubic sling were identified bilaterally and then dissected up towards the pubocervical fascia and the retropubic space without entry into the space. They were tagged for later identification and laparoscopic extraction (Figure 1).

**Figure 2:**
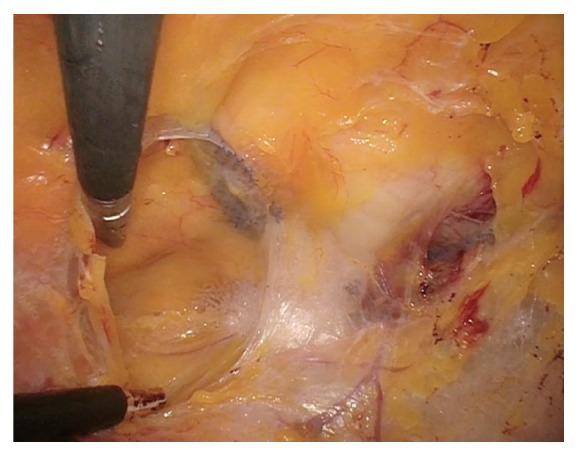
Identification of the arms of the RP sling entering through the pubocervical fascia and extending up to the abdominal wall within the retropubic space. Permission to reprint from Miklos and Moore.

**Figure 3:**
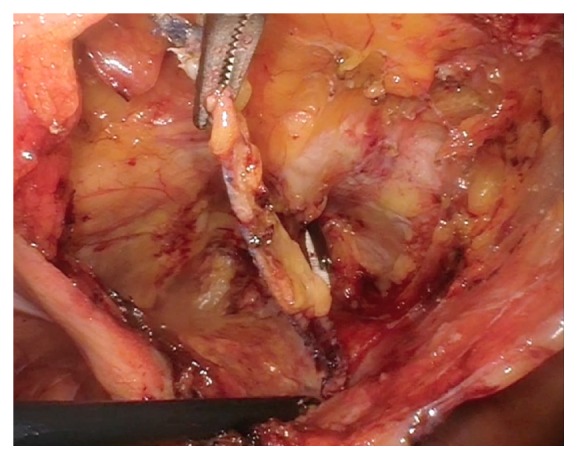
Arms of the mesh sling dissected away from the abdominal wall near the pubic rami prior to vaginal removal. Permission to reprint from Miklos and Moore.

Attention was turned to the abdominal portion of the procedure and open laparoscopy was completed through the umbilicus followed by the addition to two lateral ports and a suprapubic port. The abdomen was inspected and adhesiolysis of rectosigmoid adhesions was performed without traumatic injury to the bowel. The bladder was retrograde filled in order to visualize the superior edge of the bladder and reduce risk of injury. The vesicoperitoneal reflection was identified and the peritoneum was incised 3 cm above the border extending between the obliterated umbilical ligaments. The space of Retzius was bluntly developed until reaching the pubic symphysis. The bladder was then drained and blunt dissection was continued down into the retropubic space until reaching the pubocervical fascia. The arms of the RP sling entering through the pubocervical fascia were identified and were followed to the anterior abdominal wall on each side of the pubic symphysis (Figure 2). Using traction, the arms were dissected away from the anterior abdominal wall and released. The dissection was continued and the arms of the mesh sling were dissected away from the posterior aspect of the pubic ramus, down through the pubocervical fascia and, once free, the arms were both removed vaginally (Figure 3). Traction was placed on the vaginal arms from below to help with the dissection and removal through the pubocervical fascia. A total of 18 cm of TVT mesh sling was removed. The defects in the pubocervical fascia were repaired with 2-0 absorbable polyglycolic acid suture laparoscopically.

Her anterior wall prolapse and paravaginal defects were then addressed using interrupted placement of 2-0 nonabsorbable multifilament sutures for a bilateral paravaginal defect repair, which secured the pubocervical fascia back to the arcus tendineus fascia pelvis bilaterally with the intent to restore her anterior anatomy prior to the Burch urethropexy.

**Figure 4:**
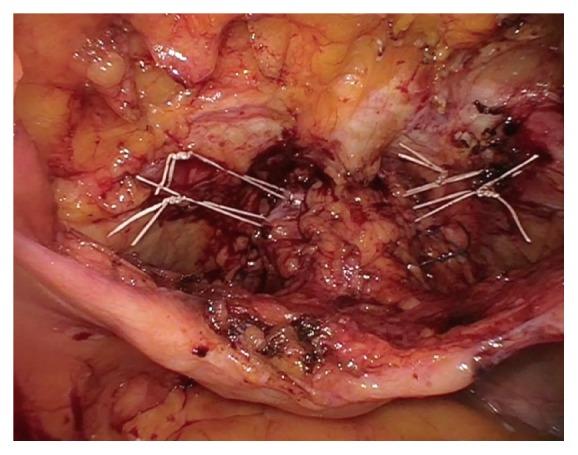
Laparoscopic Burch urethropexy with a paravaginal repair following sling removal. Permission to reprint from Miklos and Moore.

**Figure 5:**
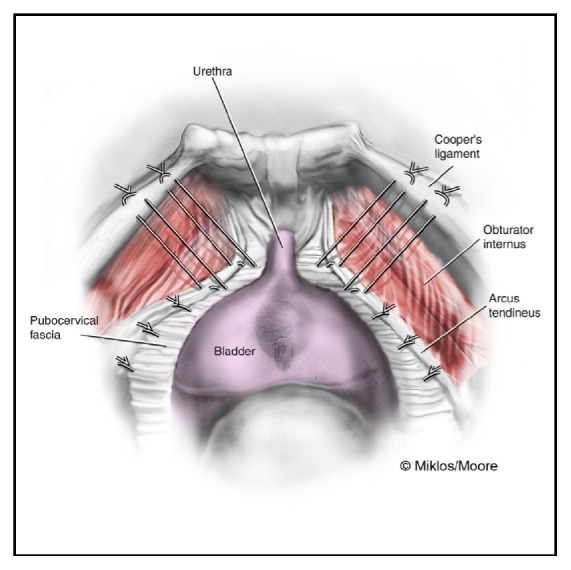
Illustration of Burch urethropexy and paravaginal repair. Permission to reprint from Miklos and Moore.

Burch urethropexy was then performed laparoscopically using CV-2 nonabsorbable polytetrafluoroethylene (Gore-Tex^©^) figure-of-eight sutures, placed bilaterally 1 cm lateral to the midurethra and at the level of the urethrovesical junction. The sutures were placed through the pubocervical fascia and carried up to Cooper's Ligament. They were tied to create appropriate bridging and tension without kinking the urethra (Figures 4 and 5). Cystourethroscopy revealed no bladder or urethral injury with bilateral ureteral jetting appreciated. The vesicoperitoneal reflection was closed laparoscopically with a running 2-0 absorbable monofilament poliglecaprone suture and was noted to be hemostatic. The abdominal cavity was inspected and found to be hemostatic, and all port sites were closed using standard technique. At the end of procedure, she underwent a posterior repair via the standard vaginal approach. The patient tolerated the procedure well without complications. Total estimated blood loss was 50 mL.

The patient was discharged on postoperative day one after successfully completing a voiding trial following removal of the Foley catheter and vaginal packing. At the time of discharge, she was given instructions for regular voiding and adequate fluid intake to prevent urinary retention. She was seen three months after surgery in the clinic and denied any further abdominal/pelvic/groin pain. In the interim, she had been treated for candida vaginitis and started on estradiol vaginal cream. She denied any symptoms of vaginal bulge, obstructive voiding, or SUI. Her postoperative IIQ-7 score was 9.5 and UDI-6 score was 25, meeting the clinically important difference cutoff or MCID [13]. The patient is now more than 6 months out from surgery, continues to do well, and is satisfied with the results. She denies any bothersome stress leakage and reports good bladder emptying. She is still not sexually active, but this is unrelated to pain which she says has fully resolved. Following her surgery, she was retrospectively enrolled in a larger study evaluating vaginal mesh complications with waiver of patient authorization as per institutional review board recommendations.

## 3. Discussion

Midurethral mesh tape slings have become a popular surgical option for stress urinary incontinence, due in large part to their ease of placement and comparable success rates compared to other anti-incontinence procedures such as the Burch urethropexy and the fascia lata slings. Even with recent reports of complications and some of the concern over the vaginal use of mesh, mesh tape slings are still considered the standard of care; they have been found to have an overall 7- to 10-year cure rate of 80% to 95%. However, given the recent public media attention concerning mesh complications, as well as the fact that if a patient has a complication with a mesh tape sling or other previous placed vaginal mesh, she may be hesitant to have more synthetic mesh placed. Unfortunately, for many of these patients, their SUI will persist or worsen after sling removal. As was performed with this patient, one option to treat refractory SUI without utilizing a mesh tape sling is via concomitant laparoscopic Burch urethropexy at the time of sling removal. As we found with this patient and subsequent other cases, performing Burch urethropexy does not increase blood loss or intraoperative complication risk. This is unlike our previous published experience with repeat laparoscopic Burch procedures following previous Burch or MMK. Although successful, we found those procedures to be extremely difficult, with increased risks of complications and blood loss secondary to the increased scar tissue found in the space [14, 15]. Based on our observations, previous RP sling placement does not seem to cause as much scar tissue in the space of Retzius as a previous MMK or Burch procedure, and removal of the sling arms is relatively straightforward.

Our case has a few unique components to highlight. Firstly, she was offered Burch urethropexy due to the persistence of her SUI that had been refractory to two mesh slings. The decision was made to proceed with Burch urethropexy rather than other nonmesh anti-incontinence procedures such as the Marshall-Marchetti-Krantz urethropexy or the fascia lata sling as the Burch has comparatively better success rates and lower risk for complications and postoperative sequelae. Furthermore, as our standard technique to midurethral sling removal involves a combined vaginal and laparoscopic approach with dissection of the retropubic space, the inclusion of a laparoscopic Burch urethropexy at time of surgery would not entail any further dissection given that the space was already open [14, 16, 17]. This patient's anterior wall prolapse could also be addressed via a laparoscopic paravaginal defect repair concomitantly.

Although there have been no studies exploring a single-stage sling removal and Burch urethropexy or any other combined anti-incontinence procedure, the general opinion has been to wait at least 6 months following sling removal to allow full healing, restoration of anatomy, and resolution of inflammation. However, this is a theoretical concern without any definitive evidence showing a difference in results. In our clinical practice, the degree of scarring in the RP space has never been severe enough to dramatically distort the anatomy or to cause any acute reactive process to degrade the urethropexy. Additionally, any defects in anterior wall support can be corrected with concomitant paravaginal defect if necessary. Once the paravaginal defect is corrected, the Burch urethropexy can be performed without concern for overcorrection of the paraurethral tissue.

Within our practice, indications for removal of a mesh sling include complaints of vaginal or pelvic pain that is reproduced with palpation of the sling, dyspareunia, dysuria, mesh erosion, obstructive voiding, or recurrent urinary tract infections. For retropubic slings, we typically offer to remove the entirety of the sling via a dual-approach surgery. For women with a symptomatic retropubic sling who opt for removal and present with refractory stress leakage without a concomitant voiding dysfunction, we offer a laparoscopic Burch urethropexy at the time of sling excision immediately following laparoscopic removal of the abdominal portion of mesh. However, for patients with transobturator slings, a laparoscopic approach is rarely indicated. Patients with a symptomatic transobturator sling and stress leakage are also offered laparoscopic Burch urethropexy at the time of sling excision. However, these patients are counseled that this would involve a separate laparoscopic procedure following removal of the vaginal portion of the transobturator mesh.

In general, prior to removing a symptomatic mesh sling, options should be provided to patients to manage stress urinary incontinence either at time of removal or at a later date. Although a repeat operation with another midurethral sling has a higher complication rate, it is commonly performed in the setting of refractory SUI and should be provided as an option to patient who would like surgical management. However, the provider must expect many patients to be hesitant to agree to this and therefore must be prepared to discuss other alternatives as well as potential complications of any approach. Laparoscopic Burch urethropexy has been an effective treatment for refractory SUI in the setting of previous failed midurethral slings that have not required removal. Based on this knowledge and reassuring results as found in this case and others like it, providers should consider concomitant Burch urethropexy for persistent SUI as a possible option for anti-incontinence at the time of sling removal.
